# Shortness of breath in a young lady, rare case report of thoracic endometriosis

**DOI:** 10.1016/j.ijscr.2022.107226

**Published:** 2022-05-18

**Authors:** Willbroad Kyejo, Allyzain Ismail, Davis Rubagumya, Rahma Bakari, Munawar Kaguta, Nancy Matillya

**Affiliations:** a Department of Family Medicine, Aga Khan University, P.O. Box 38129, Dar Es Salaam, Tanzania; b Department of Family Medicine, Premier Care Clinic Masaki, PO Box 220, Dar Es Salaam, Tanzania; c Department of General Surgery, Aga Khan University, P.O. Box 38129, Dar Es Salaam, Tanzania; d Department of Obstetrics and Gynecology, Aga Khan Hospital, P.O. Box 2289, Dar Es Salaam, Tanzania

**Keywords:** Thoracic endometriosis, Pneumohemothorax, Pleurodesis, Shortness of breath, Case report

## Abstract

**Introduction and importance:**

Endometrial glandular tissue can implant in the thorax of women suffering from endometriosis. The clinical presentation is depends on site of implantation. Complications include pneumothorax, pneumohemothorax or hemothorax.

**Case presentation:**

A 31 year old woman with history of infertility presented with shortness of breath and was found to have a significant right sided pneumohemothorax. Drainage was done followed by chemical pleurodesis using bleomycin with resolution of symptoms on her follow up.

**Clinical discussion:**

Thoracic endometriosis tend to present with chronic or sub-acute symptoms which are non-specific symptoms leading to late diagnosis. Video Assisted Thoracoscopic surgery offer both diagnostic and therapeutic in thoracic endometriosis. However in limited settings chemical pleurodesis can be carried out done to prevent recurrence of shortness of breath due to thoracic endometriosis.

**Conclusion:**

Therefore, clinical suspicion of thoracic endometriosis in evaluation of shortness of breath in a young lady with history of infertility or pelvic surgery is indispensable.

## Highlights

• Thoracic endometriosis presenting as shortness of breath
• Suspicion for thoracic endometriosis in young lady with infertility and shortness of breath
• Successful symptomatic relief via pleurodesis
• Multidisciplinary approach

## 1 Introduction and importance

Endometriosis is defined as extra uterine cavity endometrial glands and stroma [1]. It is one of the most common gynecological diseases, with pain and infertility as primary symptoms [2].

The incidence in the general population is not clear however, endometriosis is diagnosed in 30% of women with infertility and 40–50% of women who are evaluated for dysmenorrhea or pelvic pain [3].

Endometriosis most commonly involves the pelvis; particularly the ovaries, cul-de-sac, broad ligaments, and uterosacral ligaments [4]. However, endometrial tissue can be found outside of the pelvis with thoracic involvement being the commonest location [5].

In thoracic endometriosis, the following terms apply:

*Thoracic endometriosis* is defined as when endometrial tissue is identified on histological specimens (hormone receptor-positive endometrial stroma and glands) obtained from chest tube aspirate, thoracotomy, or bronchoscopy [6].
*Probable thoracic endometriosis* refers to the identification of tissue within the thorax that is suggestive but not definitively diagnostic of endometrium (e.g., stroma only or hormone receptor-negative tissue) [6].
*Thoracic endometriosis syndrome* is when one or more clinical manifestations of thoracic involvement is present (e.g., pneumothorax, hemothorax, hemoptysis, chest pain) in association with menstruation but without histological confirmation [7].
*Catamenial* refers to the occurrence of symptoms or signs that bear a temporal relationship with menses [6].

Thoracic endometriosis is a rare disease that primarily affects young women. The incidence in the general population is unknown. However, it has been reported in <1% of women undergoing pelvic surgery for suspected or known pelvic endometriosis [8]. Higher rates are reported in those with primary spontaneous pneumothorax (3 to 6%), and the highest rates are reported in those with recurrent pneumothorax or pneumothorax requiring surgery (6 to 20%) and catamenial pneumothorax (65 to 89%) [8].

Risk factors for the development of endometriosis have been described (example null parity, early menarche/late menopause, short menstrual cycles, prolonged menses, tall thin body habitus), it is unknown whether or not the similar factors increase risk for thoracic involvement [8].

Thoracic endometriosis commonly presents in young women as catamenial; pneumothorax (70 to 73%) and hemothorax (12 to 14%) [6] [7], [9]. Less common presentations include catamenial; hemoptysis, chest or scapular pain: noncatamenial pneumothorax or hemothorax, a parenchymal cavity or nodule, or diaphragmatic rupture. A high index of suspicion should exist in women of reproductive age with the above symptoms especially when they involve the right-side, present perimenstrually (usually 24 h before, and up to 72 h after menses), or the patient has a history of pelvic surgery [6] [7].

Diagnosis of thoracic endometriosis is challenging. Chest imaging with x-ray, computed tomography (CT), or magnetic resonance imaging (MRI) seldom identifies endometriosis as the cause of pneumothorax or hemothorax. Occasionally cytology can identify endometrial epithelial cells on thoracentesis or chest tube drainage of a hemothorax, however the sensitivity is low [3]. Typically, diagnosis is made through histological identification of endometrial tissue after biopsy of suspicious lesions during bronchoscopy. Bronchoscopy is recommended during or within 48 h of menstruation to improve histological yield [3].

We report a case of a woman with history of prior cyclical episodes of difficulty in breathing during periods of menstruation who was diagnosed to have thoracic endometriosis and underwent pleurodesis successfully for relief from symptoms. In this case report from Aga Khan Hospital, Dar-es-Salaam, Tanzania, we describe our approach to diagnosing and treating thoracic endometriosis with challenges faced due to financial constraints and lack of resources. This paper has been reported in line with the SCARE 2020 criteria [10]. This article has been registered with the Research Registry with identification number researchregistry7781 and can be found through the following hyperlink Browse the Registry - Research Registry.

## 2 Case presentation

31 year old nulliparous lady presented with 2 months history of gradual worsening shortness of breath which was worse during periods of menstruation and was associated with dry cough and difficulty breathing on lying flat. Her shortness of breath was relieved by keeping an extra pillow while sleeping at night. Also had a history of mild abdominal distention noted during menstruation whereby she had with heavy and painful menses lasting 5 to 7 days with a concurrent history of failure to conceive despite seeking assistance from a fertility clinic. There was no history of fever, night sweat, weight loss, lower limb swelling, awareness of her heartbeat or easy fatigability. She otherwise had no significant family history of disease, no drug allergies and did not smoke or drink alcohol. During the course of the illness she reported use of different antibiotics and herbal medication without improvement.

On examination she was alert, not pale, no lower limb edema and had stable vital signs. Respiratory system examination revealed normal chest appearance, bilateral chest expansion, trachea slightly deviated to the left side, reduced tactile vocal fremitus on the right side chest, stony dullness percussion note on right inframammary and intermammary region and absent breath on corresponding side. The left side of chest was normal. Per abdomen revealed mild distension, so scars, no obvious mass palpable nor tenderness with positive shifting dullness and rest of systemic examinations were normal.

Was initially worked up in the family medicine outpatient department and referred to the gynecology department due to suspicion of Meigs syndrome due to findings of right sided pleural effusion on X-ray and abdominal ascites with ovarian enlargement on ultrasound. Baseline laboratory investigation results are as in Table 1.

**Table 1:** Baseline laboratory tests results.

Test	Result	Range
Hemoglobin	(13.1) g/dl	(13.2–16.6) g/dl
CA 125-serum	(267.9) Umol/ml	(<46) Umol/ml
CEA-serum	(0.608) ng/ml	(0–2.5) ng/ml
AFP	(1.58) Umol/ml	(0–5.8) Umol/ml

**Fig. 1:**
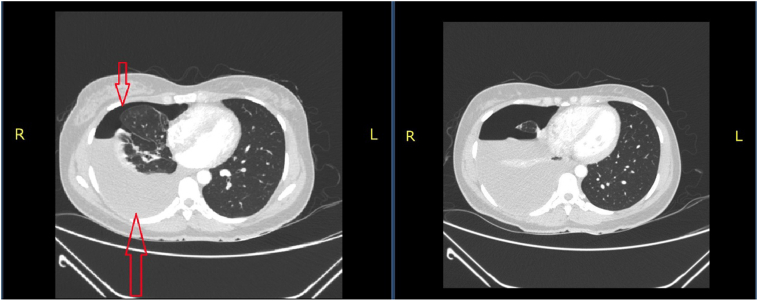
CT scan of the chest axial views - pneumohemothorax indicated by red arrows. (For interpretation of the references to colour in this figure legend, the reader is referred to the web version of this article.)

**Fig. 2:**
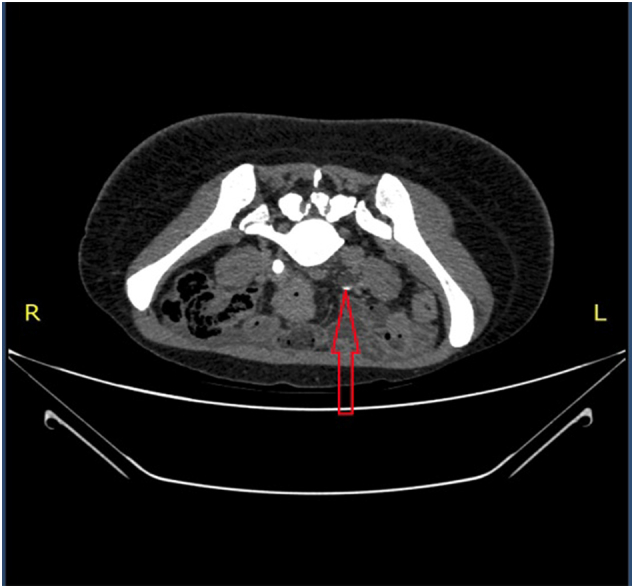
CT scan of the abdomen with IV contrast in prone position – bilaterally enlarged ovary with masses within and distal left ureteric narrowing.

In the gynecology department was reviewed by a consultant gynecologist and discussion with consultant radiologist recommended computed topography (CT) scan of the chest and abdominopelvic which suggested right moderate pneumohemothorax with partial collapse of the right lower lobe (Fig. 1), bilateral enlarged complex ovarian masses with moderate left hydroureteronephrosis due abrupt tapering of the distal ureter (Fig. 2).

General surgery team was involved and due to worsening respiratory distress chest tube for drainage was inserted on right side of chest which relieved the distress and samples of pleural fluid were taken. Results of the sample analysis revealed an acute inflammatory exudate containing abundant red blood cells and neutrophils.

The patient was consented for diagnostic laparoscopy to be carried out by consultant general surgeon and gynecologist for tissue biopsy and possible resection of ovarian mass however was converted to explorative laparatomy due to extensive adhesions, endometriotic vesicles and poor visualization. Prior to conversion to laparotomy the urologist was involved and endoscopic double J stent was inserted into the left ureter to relive left ureteric narrowing as well as to ease visualization of ureter during laparotomy so as to minimize risk of iatrogenic ureteric injury.

**Fig. 3:**
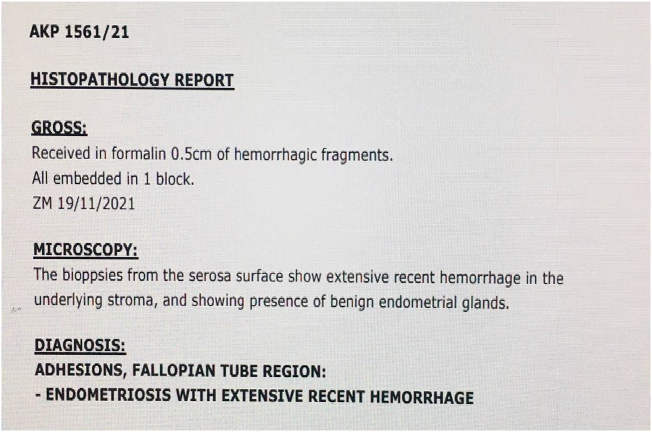
Histology report from biopsy of ovarian masses and fallopian adhesions.

Abdomen was opened via sub umbilical midline incision with findings of hemorrhagic ascites, distorted pelvis with extensive adhesions and visible endometriotic vesicles suspicious of endometriosis. The pouch of Douglas was obliterated with endometriotic vesicles and fallopian tubes were distorted bilaterally with fimbria attached to the small bowels. Only the anterior portion of uterus was visualized. Bilateral multiple ovarian masses were seen largest measuring about 2 cm. Liver, kidney, urinary bladder and spleen appeared normal. Tissue biopsy from adhesions around fallopian tube was taken and adhesiolysis was done with histology revealing endometriosis (Fig. 3).

**Fig. 4:**
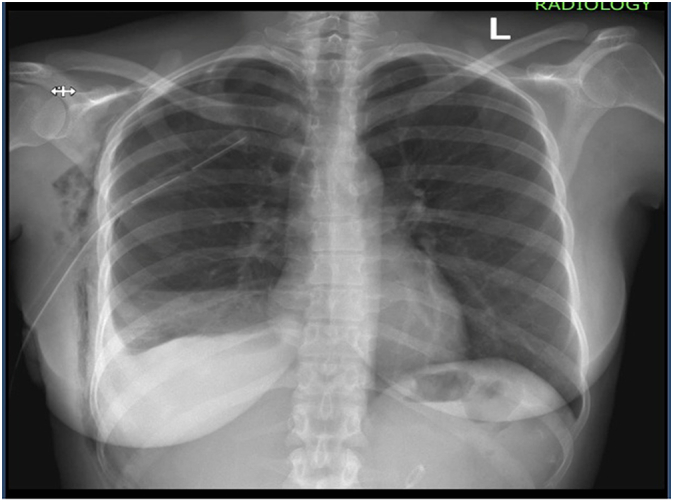
Control X-ray following chemical pleurodesis revealing mild right-sided effusion.

Post-operative patient was kept on antibiotic, intravenous (IV) Ceftriaxone 1 g every 12 h for 3 days and analgesia IV Paracetamol 1 g every 8 h for 3 days and intramuscular Pethidine 100 mg every 12 h for 1 day. In view of findings of extensive abdominal endometriosis, thoracic endometriosis was suspected as the cause of initial respiratory distress however due financial constraints and lack of expertise patient could not undergo bronchoscopy nor video assisted thoracoscopy. Hence decision to carry out chemical pleurodesis for symptomatic relief with bleomycin was done successfully (Fig. 4). Patient was discharged on 4th day post admission with minimal pain at incision site, no difficulty in breathing, no cough and scheduled to be followed up in the outpatient clinic with initiation of symptomatic treatment to reduce pain and heavy bleeding during menses due to endometriosis.

At subsequent follow up visits at day 7, day 21, 6 weeks and 3 months patient had returned to daily activity with no episodes of difficulty in breathing and reduction in amount of volume of bleeding during menses. However she still complained of painful menses more so at the lower abdominal region. Of note ureteric stent were removed on day 21 post-surgery with subsequent imaging revealing resolution of hydronephrosis with no obvious ureteric obstruction.

## 3 Discussion

Thoracic endometriosis tend to present with chronic or sub-acute symptoms which are non-specific symptoms leading to late diagnosis [10]. Our case involve symptoms despite 2 months of evaluation and management at peripheral centers [6] Despite being rare, history of difficulty in breathing, symptoms worsening with menses, dysmenorrhea and infertility should raise suspicion as it was in our case [11].

Thoracic endometriosis comprises of four different clinical categories including; pneumothorax, haemothorax, haemoptysis and pulmonary nodule according to the tissue in which it is predominant [6]. A study done in Turkey looking into 110 cases diagnosed as Thoracic Endometriosis; 73% pneumothorax, 14% hemothorax, 7% hemoptysis and nodule was 6% of cases. 85% of pleural type endometriosis is seen in right hemothorax leading to pneumothorax or pleural effusion as well as symptoms of dyspnea and chest pain [11]. The aforementioned were also found in our case, stressing the significance of clinical findings in suspecting rare conditions.

It has been postulated in the literature that thoracic endometriosis is underreported, especially when presenting as pneumothorax. Cases are often misclassified as spontaneous pneumothorax, likely due to the under recognized nature of the disease. Only after multiple recurrences or development of additional symptoms are patients finally given a diagnosis of thoracic endometriosis. Interestingly, the presence of intrathoracic endometrial implants has not fully been explained, but there are a few leading theories [3].

There are different theories for the formation of thoracic endometriosis. In the pleural type thoracic endometriosis; local metaplasia of cholemic epithelium (metaplasia theory), trans diaphragmatic pass of endometrium tissue via abdomen and diaphragmatic fenestrations from uterus and fallopian tubes (retrograde menstruation) and its implantation into the thorax cavity has been given as reasons [11].

In parenchymal type thoracic endometriosis; haematogenous expansion of endometrium tissue after surgical operations such as curettage and caesarean and micro embolization with a mechanism similar to pulmonary embolism was held responsible [11]. This process would be aided by either congenital or acquired diaphragmatic defects and could help explain right sided predominance. It is very likely the full explanation relies on a combination of these theories or a yet to be described mechanism.

Cytology examination of the pleural fluid is rarely useful [12] which is similar in our case study where it was nondiagnostic value.

With the evolution of minimally invasive procedures such as bronchoscopy and Video Assisted Thoracoscopic surgery offer both diagnostic and therapeutic in Thoracic endometriosis in more than 60% of the cases with the incidence of morbidity and mortality [11].

Concerns however the benefit of bronchoscopy is limited in lesions that are localized peripherally in parenchymal type [11].

Chest tube drainage alone without chemical pleurodesis was seen to be ineffective and showed high levels of reoccurrence [3]. As with our case chest tube drainage was kept for 3 days, following day 4 chemical pleurodesis was done to prevent recurrence. Repeat X ray showed successful drainage of right sided pleural effusion and follow up 3 weeks later revealing no residual collection.

## 4 Conclusion

Thoracic endometriosis should be considered in evaluation of shortness of breath in young female patients with history of infertility, dysmenorrhea and pelvic surgery. Prompt management is crucial, and depending with the available resources, multidisciplinary approach is of paramount importance.

## Abbreviations

AFP: Alpha Feto Protein
CT: computed tomography
IV: intravenous

## Patient perspectives

I had visited multiple centers and got antibiotics which didn't help me but once I was told of my condition I was surprised. I thought I had a lung cancer.

Staying with a chest tube for 4 days was uncomfortable but at the end of all treatment I felt much better and haven't had difficulty in breathing since.

## Consent

Written informed consent was obtained from the patient for publication of this case report and accompanying images. A copy of the written consent is available for review by the Editor-in-Chief of this journal on request.

## Ethical approval

Case study is exempt from ethical approval in my institution.

## Sources of funding

No funding was provided for research.

## Author contribution

W.K: Study conception, production of initial manuscript, collection of data

A.I: Production of initial manuscript, revision of the manuscript, proofreading

D.R: Revision of the manuscript, proofreading

R.A: Production of initial manuscript, collection of data

M.K: Revision of the manuscript, proofreading

N.M: Study conception, revision of the manuscript, proofreading

## Guarantor

Dr. Nancy Matillya, Family Medicine Consultant, Aga Khan University.

## Registration of research studies

1. Name of the registry: RESEARCH REGISTRY.
2. Unique identifying number or registration ID: researchregistry7781.
3. Hyperlink to your specific registration (must be publicly accessible and will be checked): Hyperlink.

## Declaration of competing interest

No conflicts of interest.
